# Comment on “A 10‐Year Longitudinal Study of Muscle Morphology and Performance in Masters Sprinters” by Hendrickse et al. – the authors reply

**DOI:** 10.1002/jcsm.70072

**Published:** 2025-09-24

**Authors:** Paul Hendrickse, Hans Degens

**Affiliations:** ^1^ Lancaster Medical School Lancaster University Lancaster UK; ^2^ Department of Life Sciences, Institute of Sport Manchester Metropolitan University Manchester UK; ^3^ Institute of Sport Science and Innovations Lithuanian Sports University Kaunas Lithuania

We thank Lin and colleagues [1] for their interest in our recently published 10‐year follow‐up study on changes in performance and muscle morphology in masters sprint athletes [2]. We agree that age‐related decrements in mitochondrial capacity correlate with reductions in performance in ageing people and that sleep disruption and endocrine changes, such as a testosterone decline, may underlie the decrease in mitochondrial capacity and exercise performance in ageing.

We contend, however, that impaired mitochondrial function does not directly impact sprint performance or the force output during a maximal isometric contraction as these activities rely on anaerobic, rather than aerobic metabolism [3, 4]. Although there is no direct link between muscle oxidative capacity and sprint performance, an increased electron flux and leakage from the mitochondrial electron transport chain during ageing could increase oxidative stress in the muscle cells of masters sprinters [5] that via oxidative modifications of actin and myosin could impair power generation and hence sprint performance [6]. As it has been reported that regular resistance exercise did not alter the fraction of electrons leaking to reactive oxygen species in the muscle of older people [5], we suggest that—in spite of regular resistance training—the loss of power and sprint performance we observed over 10 years in masters sprinters is most likely the result of such modifications in myosin and actin and not mitochondrial dysfunction per se [2]. While sleep deprivation and hypogonadism are detrimental to skeletal muscle health [7, 8], it is unlikely that they have a direct effect on muscle function and performance.

To illustrate our argument, one could use a thought experiment; if somehow mitochondrial function, sleep disruption and/or low testosterone were rectified in these athletes, it is unlikely that power and sprint performance would recover immediately, whereas if myosin and actin structure were restored, power and performance would likely return even if mitochondrial function, sleep status and/or testosterone levels remained the same. The distinction is that of direct determinants of muscle function and sprint performance (e.g., myofibrillar modifications) from those that precipitate (e.g., hypogonadism, sleep deprivation and mitochondrial dysfunction) poorer muscle function and sprint performance; a distinction between ultimate and proximate causes that is often not recognised.

Therefore, while it is indeed interesting to measure mitochondrial function, hormonal status and sleep quality alongside muscle function in ageing, it adds little to our understanding of what parameters per se affect muscle function in older age. They may, however, be ultimate causes that over time precipitate the proximate cause(s) (e.g., myofibrillar and/or excitation‐contraction dysfunction) of decreased muscle power and sprint performance in the absence of overt muscle fibre atrophy and capillary loss. Carefully designed in vitro and in vivo studies may elucidate the mechanisms by which these ultimate factors could contribute to impaired muscle function in older age.

## Conflicts of Interest

The authors declare no conflicts of interest.
